# Long-Term High-Density Extracellular Recordings Enable Studies of Muscle Cell Physiology

**DOI:** 10.3389/fphys.2018.01424

**Published:** 2018-10-09

**Authors:** Marta K. Lewandowska, Evgenii Bogatikov, Andreas R. Hierlemann, Anna Rostedt Punga

**Affiliations:** ^1^Department of Neuroscience, Clinical Neurophysiology, Uppsala University, Uppsala, Sweden; ^2^Department of Biosystems Science and Engineering, ETH Zürich, Basel, Switzerland

**Keywords:** muscle, spikes, spike analysis, physiology, microelectrode array (MEA) chip, skeletal

## Abstract

Skeletal (voluntary) muscle is the most abundant tissue in the body, thus making it an important biomedical research subject. Studies of neuromuscular transmission, including disorders of ion channels or receptors in autoimmune or genetic neuromuscular disorders, require high-spatial-resolution measurement techniques and an ability to acquire repeated recordings over time in order to track pharmacological interventions. Preclinical techniques for studying diseases of neuromuscular transmission can be enhanced by physiologic *ex vivo* models of tissue-tissue and cell-cell interactions. Here, we present a method, which allows tracking the development of primary skeletal muscle cells from myoblasts into mature contracting myotubes over more than 2 months. In contrast to most previous studies, the myotubes did not detach from the surface but instead formed functional networks between the myotubes, whose electrical signals were observed over the entire culturing period. Primary cultures of mouse myoblasts differentiated into contracting myotubes on a chip that contained an array of 26,400 platinum electrodes at a density of 3,265 electrodes per mm^2^. Our ability to track extracellular action potentials at subcellular resolution enabled study of skeletal muscle development and kinetics, modes of spiking and spatio-temporal relationships between muscles. The developed system in turn enables creation of a novel electrophysiological platform for establishing *ex vivo* disease models.

## Introduction

Skeletal muscle cells are excitable, multinucleated cells that generate electrical signals as a result of depolarization and, once mature, contract. Developing murine primary myotubes have been shown to contract in culture, independently from innervation, as a consequence of spontaneous variations of intracellular Ca^2+^ levels (Grouselle et al., 1991; Cognard et al., 1993; Lorenzon et al., 2002). The corresponding flux of ions results in spontaneous changes in muscle membrane potential and accompanying contractions (Powell and Fambrough, 1973). Embryonic nicotinic acetylcholine receptors (nAChR) are evenly distributed on the membrane of myotubes, and opening of these receptors occurs spontaneously in non-innervated myotubes (Jackson et al., 1990; Franco-Obregon and Lansman, 1995). After the fusion of myoblasts into myotubes, nAChRs are important both for their localization at the cell membrane as well as for their own activity; persistent autocrine release of an endogenous AChR agonist in myotubes sustains non-innervated muscles (Bandi et al., 2005). One methodological concern with myotube cultures is that, once active, cells tend to detach from the surface of culture dishes due to strong contractions, making prolonged myotube culture unwieldy (Cognard et al., 1993; Bandi et al., 2005). The beginning of prominent contractions, however, marks the true beginning of a mature cell culture that resembles physiological function in the body. It is not until an age of about 14 days that myotubes become highly developed with well-organized A- and I-bands and Z-lines along with well-organized mitochondria, and the majority of myotubes contracts in unison (Nag and Foster, 1981). Further, it may not be until almost 1 month *in vitro* that the formation of the excitation-contraction apparatus is fully developed (Flucher et al., 1994; Das et al., 2009).

In this work, we established a primary culture of mouse myoblasts that fused into myotubes on a high-density microelectrode array that enabled us to monitor electrical signaling between cells for more than 1 months. The microelectrode array chip featured 26,400 platinum working electrodes over a 3.85 × 2.10 mm^2^ area, thus providing subcellular resolution, as well at 20 kHz sampling, which enables resolving cellular action potentials or “spikes” (Ballini et al., 2014; Müller et al., 2015). We observed that, once mature, the myotubes synchronized so that their contractions could be observed electrically across the entire chip. Developing and then mature cultures were monitored on a daily basis. Spike origins were identified using timing information in two different ways: (1) Spike sorting and principal component analysis (PCA) on electrodes of interest were used to make detailed maps of spike propagation across the chip. (2) Raster plot analysis was used to identify pieces of tissue that reproducibly spiked together as well as spikes propagating in different directions. Our established electrophysiological model and methods will enable the *ex vivo* study of muscle diseases at high temporal and spatial resolution. Importantly, this could also, at least in part, offer a substitute model for *in vivo* animal studies in neuromuscular disorders.

## Methods

### Cell culture

All experiments were approved by the Uppsala Animal Ethical Committee under animal license C97/15 and follow the guidelines of the Swedish Legislation on Animal Experimentation (Animal Welfare Act SFS 2009:303) and the European Communities Council Directive (2010/63/EU). Limb muscles of post-natal mice (P1-P3) were isolated in accordance with Swedish federal laws on animal welfare using a procedure modified from Blau (Springer et al., 2002). Pups were sacrificed by decapitation, skin was removed, and all four limbs were removed and placed into sterile HBSS. Muscles were cleaned from bones and adipose tissue, and then minced into small pieces. These muscle pieces were then enzymatically digested for 1 h in 1 mg/mL collagenase/dispase solution (Sigma-Aldrich), activated using 2 mM CaCl_2_, and mechanically dissociated (Musarò and Carosio, 2017). Cells were pre-plated on uncoated T75 flasks for 1 h, and then non-adherent cells were replated onto new collagen-coated flasks. Cells were kept in F-10 based medium (F-10, Gibco, supplemented with 20% FBS, Gibco, 2.5 ng/mL FGF, Sigma-Aldrich, and 1% penicillin/streptomycin), which is preferable for myoblast survival. Every few days, after achieving about 70% confluence, cells were washed with PBS and detached 0.05% trypsin-EDTA and split. If many fibroblasts were observed, preplating was employed prior to plating in the new flasks.

After enriching for myoblasts in this manner for about 2 weeks, 100 μL droplets with a density around 250–300 k myoblasts per mL were plated onto high-density microelectrode array (HD-MEA) chips, which had previously been sterilized and treated with polyethyleneimine for 1 h and mouse laminin for 1 h. Cells were kept in proliferation medium for another 2 days and then switched to differentiation medium (DMEM, Gibco, containing 5% horse serum and 1% penicillin/streptomycin). Cells were kept in differentiation medium for the remainder of the time, and the medium was changed twice per week.

### Microelectrode array chip

The detailed information about design, fabrication, and characterization of the high-density microelectrode array chip can be found elsewhere (Ballini et al., 2014). The relevant features for the present work are as follows: 3.85 × 2.10 mm^2^ array of 26,400 bright platinum working electrodes with a pitch of 17.5 μm; 1024 reconfigurable readout channels; on-chip filtering, amplification and digitization at 20 kHz, on-chip counter electrode, and 32 stimulation units. Prior to introduction of cells, and in order to decrease electrode impedance, electrodes were electrochemically deposited with Pt-black (Frey et al., 2010). This caused the smooth, slightly recessed electrodes to become rougher and to protrude slightly.

### Electrophysiological recordings

Spike activity was recorded starting from several days after initial plating onto the chips, once cell fusion was already taking place. Initial examinations of activity were performed by using a series of 26 random configurations, each recording from 1,024 different electrodes per configuration for 20–60 s, in order to sample the chip (26 × 1,024 = 26,624, although some electrodes were not sampled while others may have been sampled several times) in an unbiased manner. When regions of interest were found, for example electrodes containing high amplitude spikes (≥1,000 μV), new readout configurations were created and used to better characterize activity of interest.

In order to create triggered spontaneous profiles, electrodes that recorded the largest spikes (often exceeding 2 mV peak-to-peak) in different regions on a given chip were selected as electrodes of interest. These electrodes of interest (usually 10–20) were kept fixed in all configurations, while the remaining 1,000 channels were routed to random electrodes. Twenty-six to thirty-two such configurations were created in order to effectively sample the entire chip, again with individual recordings lasting from 20 to 60 s each (Lewandowska et al., 2015).

### Data analysis

Data analysis was performed in Matlab using custom software. Spike detection and rudimentary spike sorting were performed offline.

#### Spike detection

Both positive and negative spikes exceeding a threshold of 6 SD of the noise, and whose derivatives exceeded 4 SD of the noise, were first identified and isolated and then combined into complex spikes. Proximity between adjacent spikes was checked to ensure against double counting, and then spikes were realigned.

#### Spike sorting

Spikes were sorted using PCA in Matlab. The number of groups and their starting positions were chosen by hand. The built-in k-means algorithm was then used for clustering. Groups could then be combined, based on similarity in shape, or template matching was employed (see below).

#### Triggered spontaneous scans

Triggered spontaneous profiles were achieved by spike sorting all signals observed on an electrode of interest (see above) over many configurations. One of the spikes (the most commonly encountered shape) was then chosen. Using the timing information associated with this spike, traces from all other configurations on all of the other electrodes were then cut out within a chosen window ±2 to ±5 ms, with a trigger provided by the spiking of the cell of interest.

#### Template matching

Template matching was used for some parts of the analysis in order to most effectively find all spikes matching a chosen profile, and to extract optimal timing information. Following PCA, a template was created for each group by averaging together all of the spikes in the group. The cross correlation between the original signal and the most common spike's template was then created and compared with the autocorrelation of the spike template. A ±5% tolerance was allowed for signal matching. This procedure generally resulted in a higher number of matches to the template while discarding poorly matching spikes resulting from the PCA.

#### Spike propagation profiles

Raster plots were converted into histograms by choosing an appropriate bin width, which could be used to distinguish different adjacent synchronized spikes from one another, 10–20 ms. These timing propagation profiles were then spike-sorted as described before by using the histogram shape as the spike. Again the k-means algorithm was used for clustering. Results were examined manually, and similar patterns were grouped together. In this manner it was possible to find a number of distinct propagation profiles within a given culture as well as the frequency of each kind of spike.

#### Jitter analysis

An electrode, which was held fixed over many subsequent configurations, served as the zero point of jitter for a given spike, and the jitter (standard deviation of the timing, measured in μs) was calculated for all other electrodes in all other configurations. This was done for several different electrodes in different parts of the same chip.

### Immunocytochemistry

Detailed protocols for fixation and staining of the cells have been described previously (Lewandowska et al., 2015). Briefly, medium was removed, and cells were washed with PBS. Cells were then fixed in 4% paraformaldehyde for 15 min, and washed with cold PBS. Cells were permeabilized with 0.25% Triton X-100 in PBS for 10 min and then washed. Unspecific binding was blocked by incubating cells in 1% BSA in PBS for 30 min. Cells were then incubated with primary antibodies (desmin, ab32362, Abcam; sarcomeric α-actinin, ab9465, Abcam) overnight at 4°C, washed, and then incubated with secondary antibodies (goat anti-mouse IgG AlexaFluor 488, A-11001, ThermoFisher and donkey anti-rabbit IgG AlexaFluor 555, ab150074, Abcam) for 1 h. After washing again, the cells were incubated with DAPI for 1 min, rinsed with PBS, and left in PBS at 4°C. Imaging was done using a Nikon Eclipse LVDIA-N microscope.

## Results

**Figure 1:**
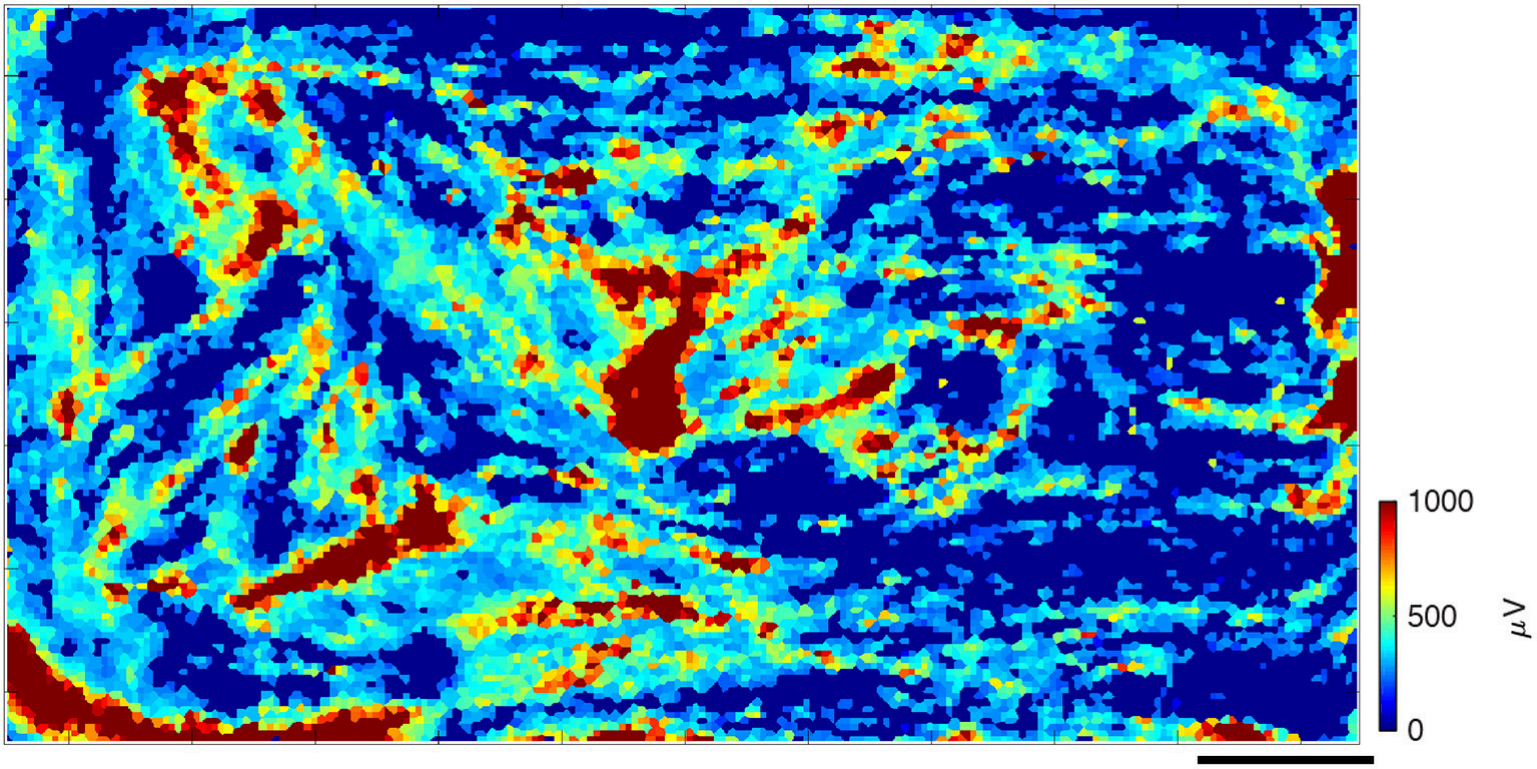
Spontaneous spiking profile, shown as a heat map of spike amplitudes after about 1 month in culture. Every square represents an electrode, whose color corresponds to the spike amplitudes that were recorded, averaged over the data-taking period. Large red “islands” of high-amplitude spikes are surrounded by lower-amplitude spiking filaments that connect them. In dark blue areas, no spikes were recorded. Scale bar is 0.5 mm.

We successfully cultured primary skeletal muscle cells on HD-MEA chips for over 60 days and observed their assembly into muscle fibers and their maturation into contracting, organized pieces of tissue. After 1–2 days in differentiation medium, myoblasts were seen fusing into myotubes. As early as 4–7 days *in vitro*, we observed isolated contractions of myotubes under the microscope (data not shown). During the first few weeks of cell culture, we observed electrical activity in the form of isolated spikes or isolated “islands” of spikes (several to tens of electrodes near each other), which slowly organized into much larger spiking areas that eventually were seen over most of the chip. At ~1 month in culture, the large “islands” of high amplitude spikes were surrounded by filaments with lower spiking amplitudes that connected them, as represented in a heat map (Figure 1). There were also areas, where no spikes were recorded (dark blue; Figure 1). Daily recordings allowed us to observe the gradual evolution of spike heights over time and the changes in activation of different myotubes on subsequent days in culture (Supplementary Movie 1). Early on, spikes were detected on a small number of electrodes, but over time this number increased, most likely as the result of the continued fusion and maturation of the aneural muscle fiber network.

**Figure 2:**
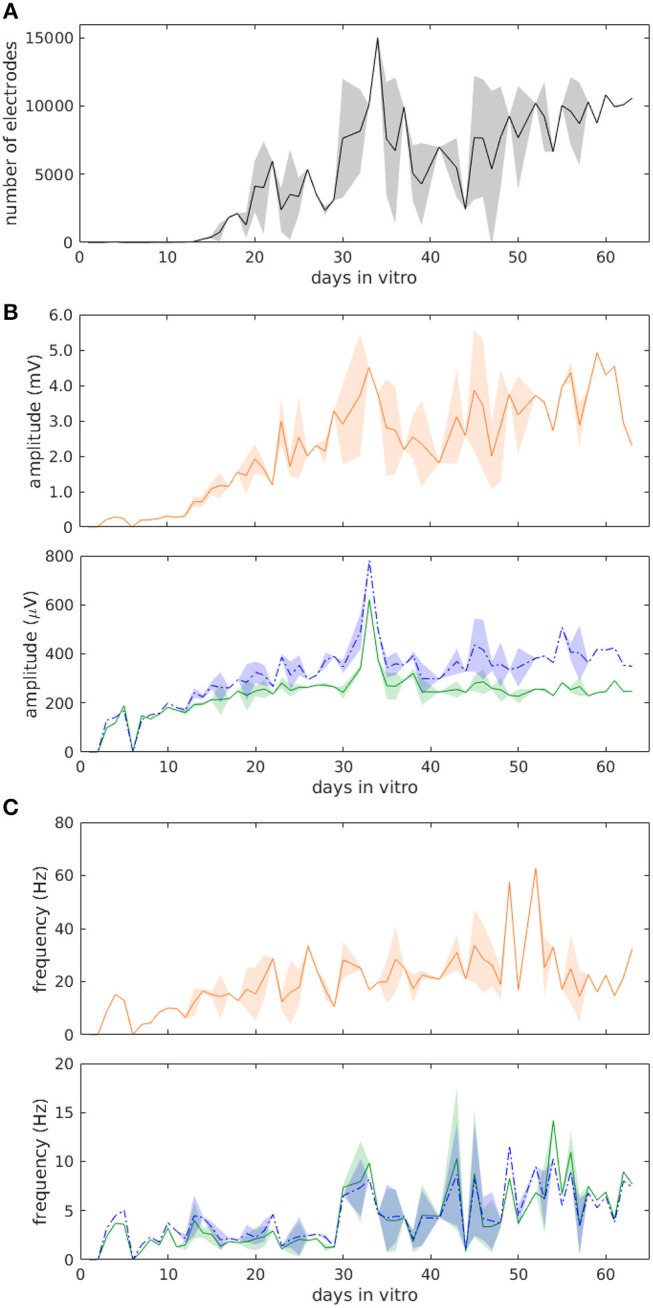
Statistics of muscle cell evolution over more than 60 days from *three* cultures. **(A)** Number of active electrodes. **(B)** Average spike height. **(C)** Average spiking frequency. Green (solid) traces show mean, blue (dotted) traces show median ± SD, and orange traces in **(B,C)** show the maximum values.

A summary of the data taken from three chips over their entire culture period is shown in Figure 2. During ≥60 days, the number of electrodes that were able to detect spikes increased over time in an approximately linear manner, but also including clear fluctuations (Figure 2A). Toward the end of the culturing period, most cultures had reached a steady state and appeared to be declining somewhat in their activity as well as in the number of electrodes that were monitoring spikes. The mean spike height, after undergoing fluctuations around day 10, leveled out to around 300 μV, while the median height leveled out to around 400 μV (Figure 2B). Additionally, the spike frequency fluctuated dramatically over this time period, although the frequency remained around a value of 5 Hz (Figure 2C).

**Figure 3:**
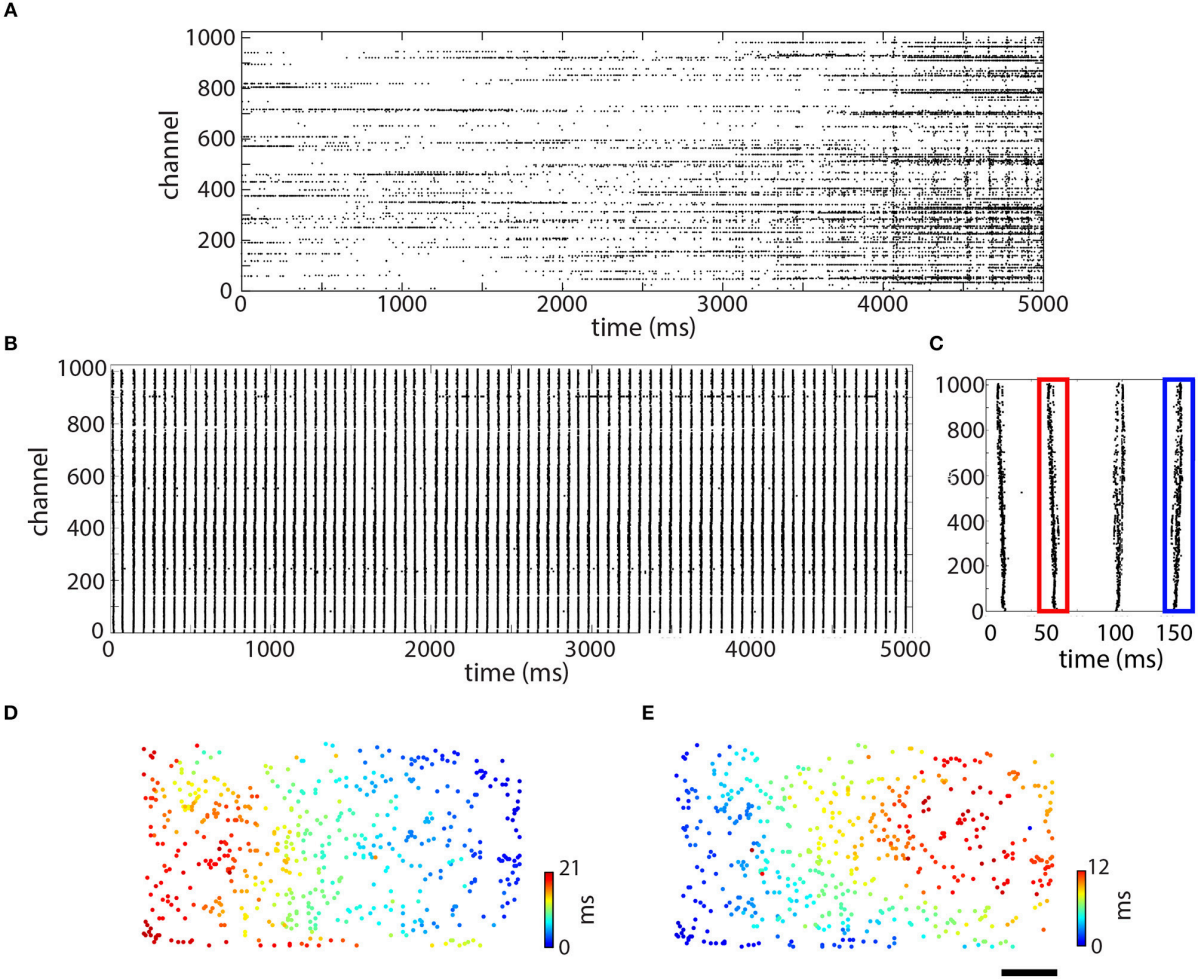
Time and space-resolved information about spiking over the culture. Raster plots showing data taken at 19 days *in vitro* (DIV) **(A)** and 31 DIV **(B)**. Each of these raster plots shows 5 s of data. **(C)** Zoom-in on first four spikes shown in **(B)**. **(D,E)** Space resolved timing information for the spikes shown in **(C)**. **(D)** Second spike, outlined in red. **(E)** Fourth spike, outlined in blue. Scale bar in **(E)** is 0.5 mm and applies to **(D,E)**.

After about 20 days in culture, the assembled myotubes had effectively formed into a piece of skeletal muscle tissue that contracted at the same rate all across the chip. Figure 3 shows raster plots from a single configuration taken from one culture after 19 (Figure 3A) and 31 (Figure 3B) days *in vitro* (DIV). We observed that different electrodes spiked somewhat randomly with respect to one another, with some displaying sparse spiking and others instead exhibiting tonic activity (Figure 3A). Between 4 and 5 s, a higher frequency of activity was observed and a couple of hundred electrodes (out of 1,024) spiked somewhat synchronously in what appeared as five or six vertical blocks. By 31 DIV (although also earlier), the spikes in the same culture had become synchronized (Figure 3B). Over the same 5 s period, there were 79 vertical lines (corresponding to a frequency of 15.8 Hz), and virtually all of the electrodes were participating, with little other activity observed. This pattern of synchronization over time was typical and observed in all cultures (*N* = 9).

Spikes propagating in different directions were also synchronized across the culture (Figure 3B), which was made evident by looking at the raster plot in greater detail. Examining the first 250 ms (4 spikes; Figure 3C), it became apparent that there is a fine structure to the “vertical lines,” which was actually different for the spikes shown. All four spikes (Figure 3C) appeared to be somewhat different from one another, although the first two were more similar to one another and the second two were also somewhat similar to one another. The timing information for the spikes outlined in red and blue is shown as a filled circle in its physical location on the chip (Figure 3D,E). In both of these plots, color conveys timing information from blue (0 ms in both plots) to red (21 ms in Figure 3D and 12 ms in Figure 3E). As can be seen, these two spikes were propagating in opposite directions on the chip but at similar speeds, around 0.2 m/s. Individual pieces of the tissue, however, spiked more quickly, ranging from 0.5 to 1.1 m/s. In Figure 3D, the spike originated on the upper right and propagated toward the lower left. In Figure 3E, the spike instead originated on the bottom left and propagated toward the upper right. Although both of these spikes were recorded in the same electrode configuration, not all of the electrodes participated in each spike.

**Figure 4:**
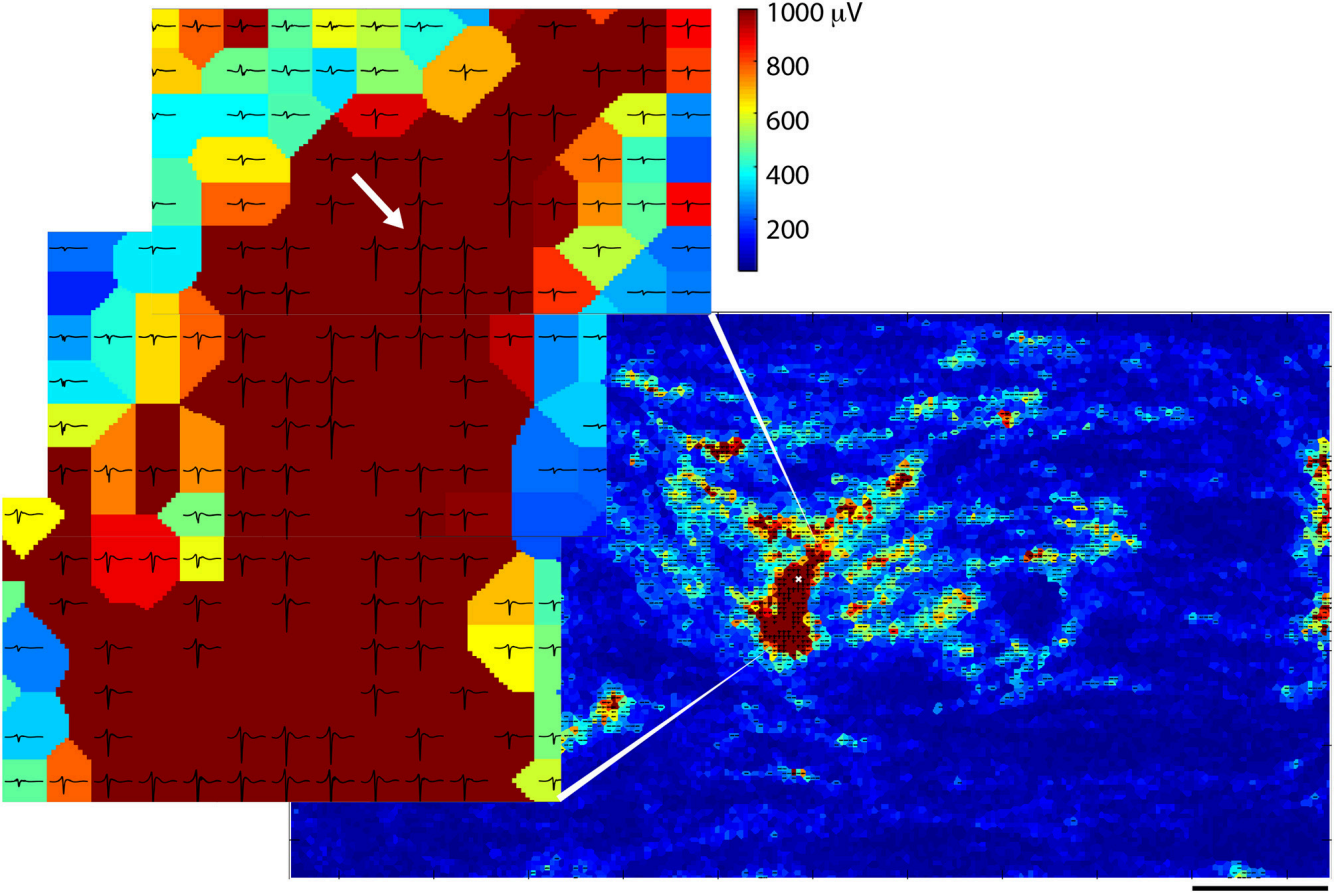
Spike sorting of spontaneous data from *one* of several spikes observed on a chosen electrode. The heat map shows average spike amplitudes and traces in which spike amplitudes exceeded 300 μV are overlaid on top of electrodes. The chosen electrode is denoted with a white “x” in the zoomed-out picture, and with a white arrow in the zoomed-in picture. All other electrodes are represented by color according to spike amplitude.

A triggered spontaneous profile (see section Methods) was used to visualize more detailed information about spike propagation across the chip. Figure 4 shows one such typical profile with the timing window opened up to the spike length of the originating spike ±2 ms. The data were taken on the same day as Figure 1 (~30 DIV) and they shared clear common characteristics. In the zoomed-in part of Figure 4, already the effects of propagation can be seen: the smaller traces on the electrodes in the upper left appear to precede the larger ones in the middle. The traces that are shown are averaged templates from all of the traces that were measured on any particular electrode during the recording session.

**Figure 5:**
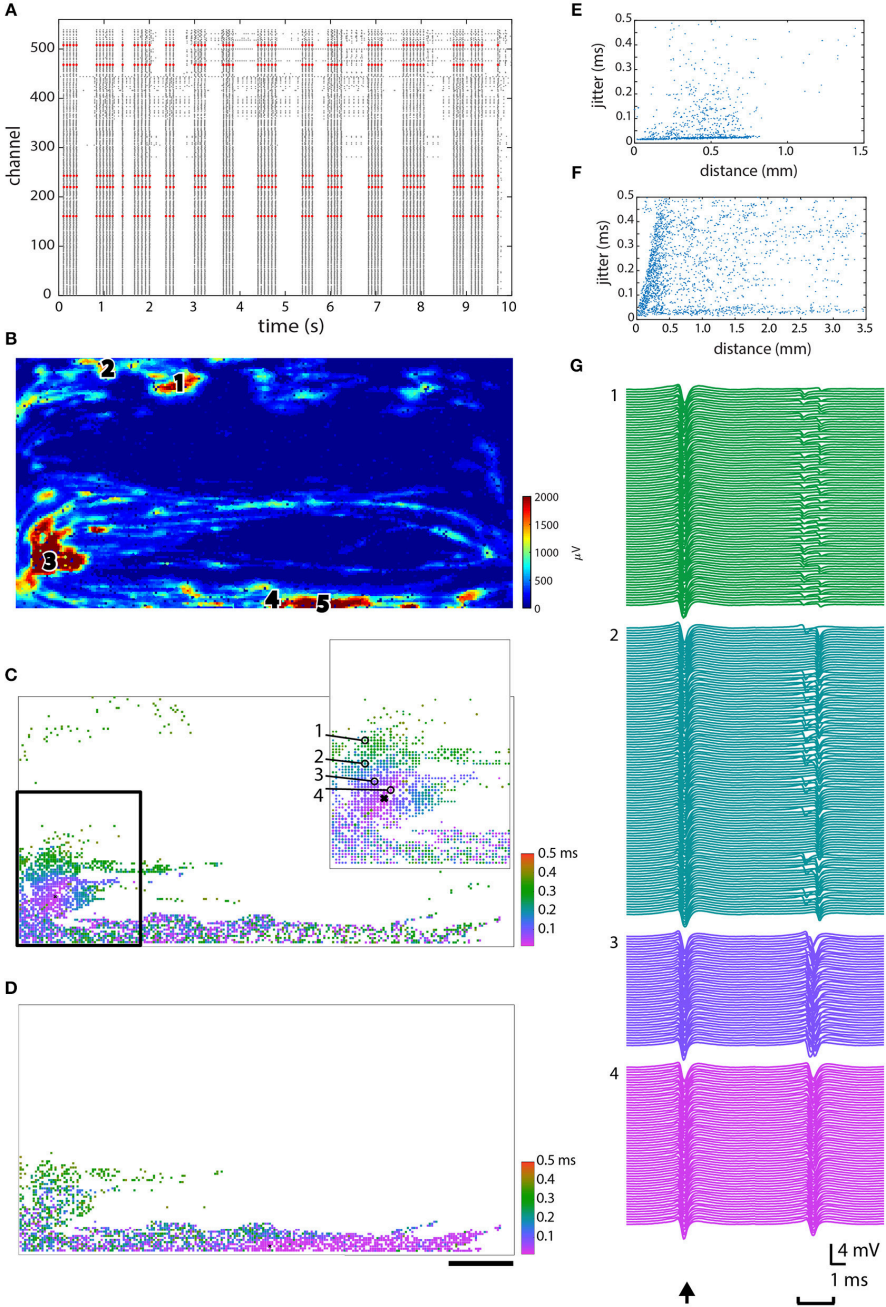
Jitter analysis. **(A)** Raster plot showing 10 s of data, with the five electrodes in high spiking areas that were chosen for further analysis plotted in red. **(B)** Spatial configuration demonstrating where each of the chosen electrodes was located. **(C,D)** The jitter on electrode 3 and 4, respectively, as indicated in **(B)**. **(E,F)** Jitter vs. distance plotted for electrodes 2 and 3, respectively, as indicated in **(B)**. **(G)** Signals from the circled electrodes (1–4) in C inset vs. the “zero point” that signals were aligned to (indicated with an “x”), to demonstrate the actual measured jitter, as shown in **(C)**. Scale bar in D is 0.5 mm and applies to **(B–D)**.

Propagation jitter was calculated in an attempt to determine which muscle tissue pieces were most directly linked to one other. The calculation of jitter was done for several different electrodes in different parts of the same chip (Figure 5). Five of the fixed electrodes were chosen for further analysis, and are shown in red in the raster plot (Figure 5A) and as pink or white circles on the heat map (Figure 5C). In this case, all five electrodes recorded the synchronized spikes—the vertical “lines”—in the culture (see Figure 3B) as visualized in Figure 5A. The jitter on two different electrodes (marked 3 and 4) is shown in Figures 5B,D. The inset in Figures 5B,B2 serve to demonstrate exactly what is meant by jitter: traces from the five marked electrodes in the inset are shown in 5B2. The trace from the electrode marked with the “x” provides the exact timing, and then traces from the four circled electrodes (marked 1–4) demonstrate the increasing jitter from pink (<50 μs jitter) up to green (250–350 μs jitter). Jitter vs. distance is plotted for three electrodes marked 2, 3, and 4 in Figure 5C in Figures 5E–G respectively. Data from the other two electrodes, marked 1 and 5, in Figure 5C showed profiles similar to Figures 5E,G and are, therefore, not shown.

**Figure 6:**
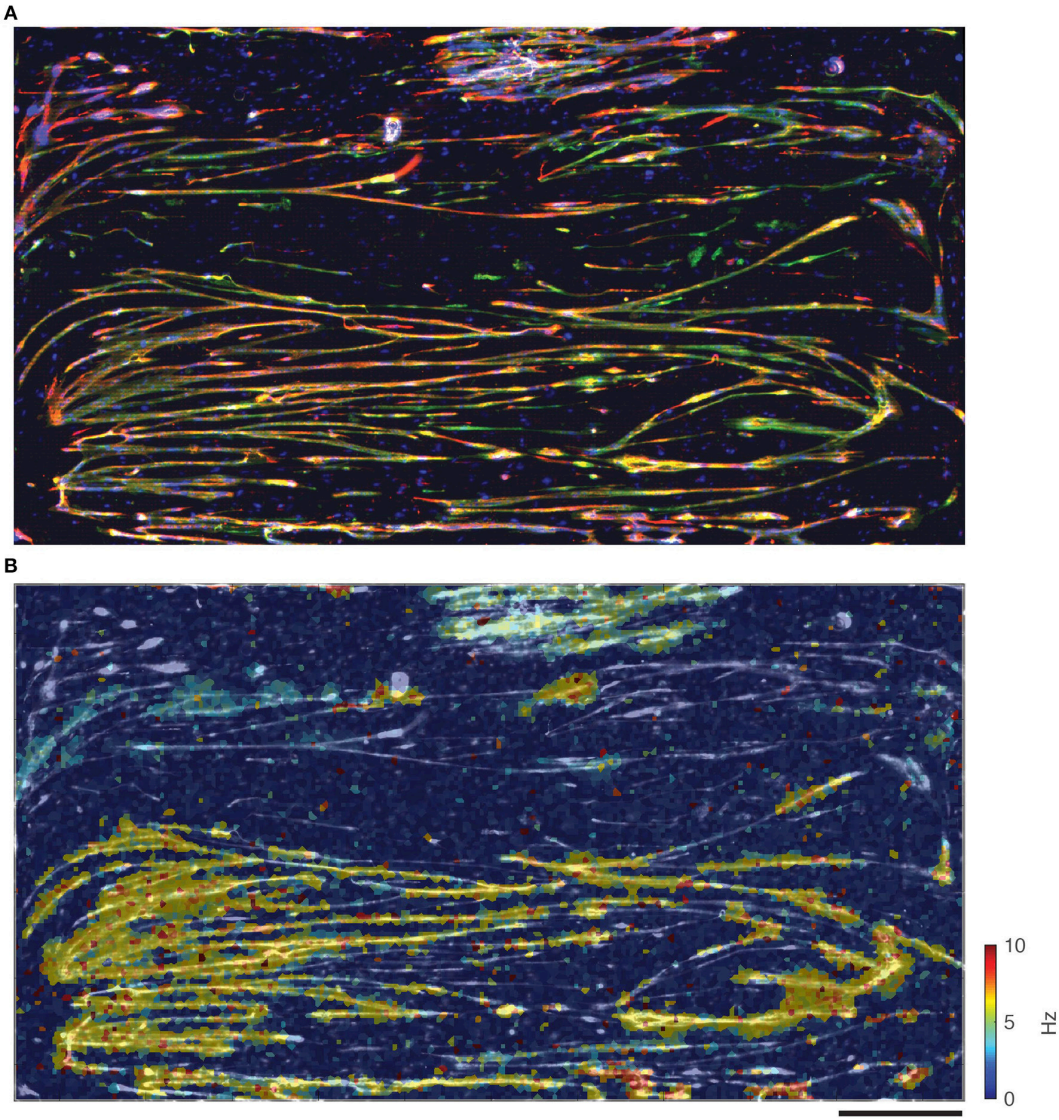
Comparison between immunostained muscle cells and electrical spike activity. **(A)** Immunostained muscle cells on the electrode area stained for desmin (red), α-actinin (green), and DAPI (blue). **(B)** Overlay of immunostained cells (in black and white) and activity (spike frequency in color). Scale bar is 0.5 mm.

At the end of the culturing period, the skeletal muscle cells on the chips were fixed and stained to determine maturity of the myotubes. Antibodies against desmin (a muscle-specific intermediate filament, which defines sarcomeres), sarcomeric α-actinin (microfilament needed for Z-line attachment), and DAPI (doubled stranded DNA) were used. One such representative staining (Figure 6A) of the entire chip area indicated good agreement between the stained muscle cells and the overlaid electrical heat map (Figure 6B). Although many single cells could be seen, most cells appeared to have fused into myotubes (Figure 6A). There were quite a few blue dots, which were probably primary myocytes that retained their satellite cell character rather than fusing into myotubes. Most of the chip surface, however, was dominated by long myotubes that stained positive for both, desmin and actinin, indicating that these myotubes indeed possessed a fully developed sarcomere and, thus, the contractile apparatus typical for skeletal myofibers.

One of the crucial findings was that electrical activity (i.e., distribution and characteristics of spikes) correlated well with morphological images obtained through microscopy. Data taken on the day of fixation (Figure 6B) was overlaid onto the image in Figure 6A (this time in black and white) to correlate electrical activity with cell locations, and shows good agreement. Although there were areas covered with cells that did not exhibit electrical activity during the data session, there were no anomalous areas of electrical activity not corresponding to cells. The fibrous structures, so often seen in the spike data, correlated very well with the myotube morphology, as observed in the microscopy images.

## Discussion

The main advantages of the developed methods presented here are the possibilities to localize extracellular signals in single skeletal myotubes, both at cellular and subcellular levels, as well as in groups of myotubes and to detect different firing patterns over a considerable time period. The tools we established have allowed us to examine muscle-cell activity in complementary ways and to observe organization of the tissue over time and space. Not only do our methods allow us to examine muscle tissue at an unprecedented level of detail, but will enable further work into disease models. This allows for studies of both, normal development of myotubes as well as disease processes over time with a resolution that can detect disease mechanisms occurring within seconds. Since very few groups have used microelectrode arrays to examine extracellular muscle signals (Langhammer et al., 2011; Rabieh et al., 2016), we have few outside sources with which to compare our results; however, it is clear that our methods can be used to study muscle tissue in great detail.

Our first electrical observations of muscle cell activity occurred after ~2 weeks *in vitro*. Of the two groups that have used skeletal muscle preparations on microelectrode arrays, one group was able to measure such signals after just 2 days *in vitro*. Their three-dimensional mushroom shaped electrodes also allowed for measurements of intracellular signals (Rabieh et al., 2016). We did not manage to detect signals at such an early time point, and we wonder whether their extracellular signals may indeed have had some intracellular components, which enabled such early observations. The only other group to use planar electrodes also observed signals after about 2 weeks *in vitro* (Langhammer et al., 2011). We found that the development we observed correlated well with the observations of others; myoblasts fused into myotubes on the second day *in vitro* and myotubes started to show rudimentary contractions after about 1 week *in vitro* (Nag and Foster, 1981; Lorenzon et al., 2002). At these early stages, however, the myotubes were not yet mature (Rabieh et al., 2016), and only entered a phase of maturation, when the fibers were fully differentiated and exhibited well organized A- and I-bands and Z-lines around 14 DIV (Nag and Foster, 1981). Our ability to start detecting signals around 2 weeks *in vitro* most likely corresponds to this important organizational milestone of the developed sarcomere.

One obvious advantage to our method was that we were able to measure extracellular signals over a long time period, up to 2 months. Most other groups report delamination of the muscle tissue upon full maturation when strong contractions begin. This tends to happen between seven and 14 DIV (Lorenzon et al., 2002; Sciancalepore et al., 2005). Some groups that report electrophysiological data from myotubes older than 2 weeks mention myoballs, i.e., balls of cells that have detached from the substrate although they continue to function electrically (Cognard et al., 1993). One group that reported contracting myotubes on a substrate after 25–90 days in culture used embryonic myoblasts for their culture and did not demonstrate electrophysiological data over this whole time period, but only at a single time point, at 4 DIV (Das et al., 2006) or 48 DIV (Das et al., 2009). What is clear from their publications, however, is that full maturation of the myotubes may not occur until almost 1 month *in vitro*. The ability to keep a culture viable and interrogable for long periods is essential for studying normal physiology and establishing disease models, allowing for interventions and their follow-up.

While we have not achieved the longest culturing times for myotubes (Das et al., 2009), this was not a key research aim. Instead, we intended to demonstrate the electrophysiological development of the muscle cell culture over such long time periods, especially with respect to mature muscle machinery. Our observation, that the muscle matured over time and then reached a steady state, was punctuated by fluctuations. We observed a high-frequency electrical-activity pattern in different parts of the culture that changed over time. Some of these fluctuations were seen in Figure 2, although they were most evident in the Supplementary Movie 1. Between days 32 and 35, for instance, there was a clear movement of myotubes away from the upper right corner of the chip. A myotube that could be seen electrically on day 58 (in blue near the middle of the chip) disappeared the next day to only reappear on day 60. There were also inexplicably quiet days (day 39), when very few muscle fibers appeared to be spiking. Such observations were not the result of inadequate sampling, but rather reflect true physiological changes occurring in the myotube culture. The origin of the spontaneous activity, observed in myotubes, are voltage-gated Na^+^ channels (Frelin et al., 1984; Brodie et al., 1989), L-type Ca^2+^ channels (Lorenzon et al., 2002; Shtifman et al., 2004) and nAChRs. The high-frequency (>1 Hz) spontaneous electrical activity in mouse myotubes results mainly from the interplay between Na^+^, Ca^2+^, and Ca^2+^-activated K^+^ currents (Sciancalepore et al., 2005). It has been suggested that the activity of the AChRs is responsible for the low frequency (<0.5 Hz) activation of the excitation–contraction cascade up to 0.45 Hz (Bandi et al., 2005).

Limitations to our study stem from the two-dimensional nature of the skeletal muscle cell culture on the HD-MEA substrate. Current approaches to tissue culture in general, and muscle cell culture in particular, are increasingly focusing on three-dimensional cultures, sometimes referred to as organoids, which better mimic the *in vivo* environment (Engler et al., 2004; Grosberg et al., 2012; Bhatia and Ingber, 2014; Uzel et al., 2016). Vascularization of muscle has been achieved *in vitro* (Levenberg et al., 2005; Carosio et al., 2013). Bioreactors and bioactuators allow force measurements to be conducted, and can even electrically or mechanically stimulate the muscle in order to increase its force output (Dennis and Kosnik, 2000; Legant et al., 2009; Donnelly et al., 2010; Hosseini et al., 2012; Sakar et al., 2012; Cittadella Vigodarzere and Mantero, 2014). While these approaches are clearly superior in their ability to reconstitute a more natural environment for better muscle cell function, they lack greatly in resolution since even micro-fabricated posts are usually several hundred microns away from one another (Legant et al., 2009; Sakar et al., 2012). In order to record APs, as we have done, three-dimensional MEAs or cuff electrodes would have to be employed, and although these do exist and continue to become more sophisticated, they also have limited resolution (100 to several hundred microns) and a very limited number of readout electrodes compared to the high-density planar array that we have used (Maynard et al., 1997; Rabieh et al., 2016; Xiang et al., 2016).

The data analysis proved to be a challenge, since microelectrode arrays are usually used to study neuronal cell cultures, and, thus, most of the signal-analysis methods are tailored for neurons. We observed a large variety of spike shapes, which ranged from short monophasic positive or monophasic negative spikes to long polyphasic muscle-unit action potentials (MUAPs) like those, observed using electromyography (EMG) *in vivo*, and everything in between (Stashuk, 2001), see the Supplementary Figure 1 for examples of spike shapes that were observed. Neurons tend to show biphasic (first negative and then positive) spikes, whose major phase is negative, allowing one to set a single threshold in the negative direction. Aside from having a characteristic shape, neuronal spikes also tend to have limited length, and neurons themselves have a refractory period that can be used to aid in spike sorting (Lewicki, 1998). However, in the case of muscle cells we observed that a given electrode picked up spikes from multiple cells or cellular compartments thus giving rise to MUAPs that were complex and could become more complex after longer culture times. This complexity of signals is expected as a result of the expanding myotube network. Rabieh et al. made similar observations and they explain these complex and changing spike shapes with an electrical model of the myotube network, which demonstrates electrical coupling between myotubes (Rabieh et al., 2016). In our case, the wealth of data, enabled by the large number and proximity of electrodes, and the complex spike shapes, made analysis fairly difficult. We could take neither length nor polarity of spikes for granted. In the case of EMG, many algorithms have been developed for the purpose of resolving complex MUAPs into individual spikes (LeFever and De Luca, 1982; LeFever et al., 1982). We decided not to pursue this angle yet, especially since our data, from so many electrodes, would take incredible computing power and time to process (EMG is usually done with one or up to several electrodes), while the results would likely not significantly enhance our understanding of the physiology.

We believe that the regions, where the largest spikes were detected, are probably attachment points of the muscle, where the cells most closely adhered to the electrode array. These tended to be in the periphery of the array and also appeared to be regions where the spikes initiated. This may be due to the large reference electrode that encircles the array and is often covered by a layer of dendritic Pt black. The roughness of the deposited Pt likely serves as a point of adhesion for the myotubes, and may have been an important ingredient in prolonging the possible culture time. However, high spiking regions in which spikes originated were also found on other parts of the HD-MEA array, as Figure 1 clearly shows. The other regions were dominated by long fibers that connected the attached muscle pieces to one another. In these other regions the spikes tended to be smaller, and their shapes were less robust and more prone to change. The effect of movement, brought about by contraction, could be expected to be the most noticeable in these regions.

In spite of our efforts, the ability to distinguish between different muscle units has not yet been achieved. After several weeks *in vitro*, most electrodes began spiking in a synchronized manner, making it difficult to tell individual units apart from one another using merely frequency as a criterion. Although it appears that spikes can start from different initiation points, those still propagate through the whole culture. We calculated the propagation jitter starting from different electrodes to see whether this would demonstrate some clear divisions between units. Jitter has been used *in vivo* as a diagnostic tool for studying neuromuscular transmission between muscles innervated by the same motor nerve (Stålberg et al., 1971). In healthy innervated muscles *in vivo*, a jitter value around 20–30 μs is considered normal, and this can increase above 80–100 μs in particular disorders of neuromuscular transmission (Stålberg et al., 1974; Weir et al., 1979). Since our muscle cells were not innervated, we were not expecting comparable jitter values, although we imagined that there might be clear delineations between areas that were more closely connected (muscle fibers or units) and other parts of the culture. In our cultures we observed areas with jitter ≤ 50 μs, and as the distance from the selected electrode increased, so too did the jitter, to around 100–150 μs. There were also small local areas with low jitter that rapidly increased to 200 μs in the surrounding tissue. We examined a rather wide range of jitter values from tens of microseconds up to milliseconds, in order to find suitable values that might result in distinctions between fibers, but it is possible that we did not investigate the correct range for our muscle fibers. It's also possible that due to the lack of innervation, there were no individual muscle units, but only a single large unit with local activity.

Ongoing work to introduce motor neurons and establish a co-culture system, as well as to electrically stimulate the muscle cells directly, is expected to aid in the further organization and maturation of the culture. Although muscle tissue undergoes a substantial amount of organization on its own, including prepatterning of AChRs, the presence of the motor axon and innervation greatly improves its organization and synchronizes its action (Sanes and Lichtman, 2001; Kummer et al., 2004, 2006). Additionally, there is plenty of evidence that mechanical as well as electrical stimulation improves tissue-engineered muscle. Elasticity, power output, excitability, and indeed functional maturation, have all been found to increase given the correct stimulation parameters (Vandenburgh, 1988; Powell et al., 2002; Moon et al., 2008; Khodabukus and Baar, 2011; Ito et al., 2014). It is our intention to stimulate our cells directly using the HD-MEA, as well as to stimulate indirectly via co-cultured motor neurons, which should also help to better assemble and organize the post-synaptic membrane (Wu et al., 2010).

## Conclusion

We cultured muscle cells from primary myoblasts to differentiated polynucleated myotubes, which we kept for over 60 days in culture, and observed their electrical activity over this entire time period. Looking at individual electrical spikes as well as average heights and frequencies on individual electrodes at subcellular resolution, we followed and documented the maturation of the myotubes and their assembly into mature contracting muscle tissue. Over time the average height of spikes observed leveled out to several hundred microvolts with a frequency around 5 Hz.

Once mature, the contracting skeletal muscle tissue spiked with a common frequency across the chip, and we observed spikes originating from different parts of the culture and propagating in different directions at various speeds, ranging from around 0.6 m/s up to about 1.1 m/s. We were able to make gross observations in single configurations, and very fine observations of spike shapes using triggered spontaneous images after spike sorting electrodes of interest. The electrical signatures of mature muscle fibers that were observed correlated well with images obtained using fluorescence microscopy.

Our future aims are to establish a co-culture system using primary motor neurons in order to establish a neuromuscular junction on the chip. Culturing neurons either on top of or beside myotubes should result in improved maturation of the myotubes as well as functional synapses between the two cell types. Our repository of tools including frequency and height analysis as well as measurements of jitter should prove very useful for this more complicated culture system. Chemical stimulation or electrical stimulation using the microelectrode array will allow us to establish connectivity maps between the two cell types.

## Author contributions

ML conceived of and performed experiments, wrote software and performed analysis, and wrote the manuscript. EB performed cell culture and experiments. AH provided technology and helped to edit the manuscript. AP helped to conceive experiments, assisted with analysis, and helped to write the manuscript.

### Conflict of interest statement

The authors declare that the research was conducted in the absence of any commercial or financial relationships that could be construed as a potential conflict of interest.
