# Supplementary material for: Long-Term High-Density Extracellular Recordings Enable Studies of Muscle Cell Physiology
Source: Front Physiol. 2018 Oct 9;9:1424. doi: 10.3389/fphys.2018.01424 (PMC6190753; doi:10.3389/fphys.2018.01424)
Supplement: Supplementary file 1 [file Table_1.docx]

**Supplementary Figure**: Spike shapes observed on different electrodes. A. Biphasic spikes were the most commonly observed, accounting for about 60% of spikes. B. and C. Monophasic spikes were also observed where negative ones, like C, were much more common. D. More complicated biphasic-like spikes were also fairly common. E. Complicated polyphasic spikes were observed as well, on about 20% of electrodes. F. Very large biphasic spikes were also observed. Scale bar in A applies for A-E.

**Supplementary Movie**: Maturation of the muscle cell culture over two months. Amplitude and frequency information displayed as a movie over 57 DIV. Initially isolated spikes were observed on the array that slowly organized into larger structures while activity frequencies also vary as the culture matures.
