# Supplementary figures and images for: Long-Term High-Density Extracellular Recordings Enable Studies of Muscle Cell Physiology
Source: Front Physiol. 2018 Oct 9;9:1424. doi: 10.3389/fphys.2018.01424 (PMC6190753; doi:10.3389/fphys.2018.01424)

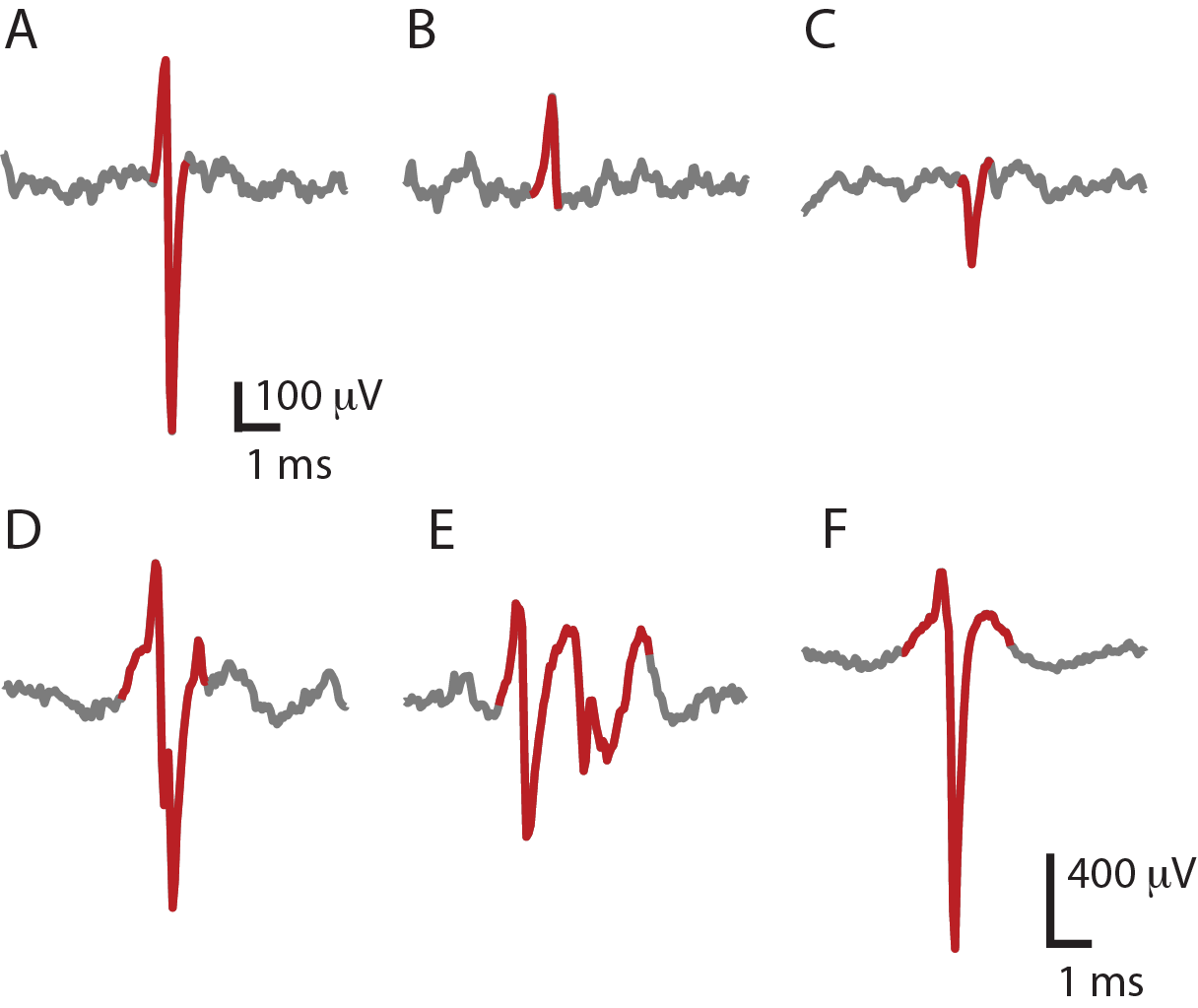

Supplement: Supplementary file 3 [file Image_1.TIF]
